# Coming from two different worlds—A qualitative, exploratory study of the collaboration between patient representatives and researchers

**DOI:** 10.1111/hex.12875

**Published:** 2019-02-18

**Authors:** Susanne Stuhlfauth, Ingrid Ruud Knutsen, Christina Foss

**Affiliations:** ^1^ Department of Nursing Science, Faculty of Medicine University of Oslo Oslo Norway; ^2^ Department of Nursing and Health Promotion Faculty of Health Science, Oslo Metropolitan University Kjeller Norway

**Keywords:** collaboration, community participation, constructivism, discourse, focus group, interprofessional relation, positioning theory, research personnel, user involvement in research

## Abstract

**Background:**

Interest in user involvement in research has increased and user involvement is increasingly seen as a prerequisite. Still, challenges in the collaboration process have been documented from both researchers' and users' perspective.

**Objective:**

By bringing together researchers and patient representatives, this study explores and describes both parties' experiences with user involvement in research as they appear through interactions in a focus group.

**Design:**

We apply a qualitative design using positioning theory as a theoretical framework.

**Setting and participants:**

Researchers and patient representatives were mixed within 2 focus groups. Positioning theory was used to guide the analysis.

**Findings:**

The discussion evolved around knowledge, equity and partnership, all related to power through constant negotiations of positions. Researchers and users ascribed various positions while discussing these topics. Various positions are seen as the result of different rights and duties in the research process. Power differences in the form of different rights and duties stand out as barriers. Being positioned as a partner was an important aspect for users in our study. Researchers assumed passive positions within the focus group, whereas users assumed active positions by expressing their wishes and needs.

**Discussion and conclusion:**

Our study indicates that positions relating to status and knowledge in the involvement process are important. The findings suggest that the positions that users and researchers assume and ascribe throughout the process are constantly changing; however, the researchers tend to have more power. More studies are needed to understand how equity is perceived in user involvement in health research.

## 1 INTRODUCTION

User involvement in research has gradually increased in Western countries over the last two decades. User involvement has broadly been defined as performing research “with” or “by” the public, rather than “on,” “about” or “for” the public.^1^ Although involving users is described as an ideal and in some countries a prerequisite for public funding, it has proved to be difficult.^2^, ^3^, ^4^

Collaboration between researchers and users in health research implies collaboration between persons with differing backgrounds, knowledge and experiences. Research indicates that one central challenge is power differences between users and researchers.^4^, ^5^ This denotes a need to further investigate the impact of differences between researchers and users and how dissimilarities may influence their perception of what involvement is and how it should be carried out.

Previous studies have focused on experiences from either users or researchers,^6^, ^7^, ^8^ but we have not found any studies that have given users and researchers within health research the opportunity to discuss experiences on user involvement. Research indicates that focusing on the interaction process is one possible way of gaining in‐depth understanding of why user involvement in research is experienced as challenging,^9^ and we thus wanted to study this interaction by bringing patient representatives and researchers together.

The foundation for user involvement is based on different rationales; an economic and consumerist discourse with the goal of cost effectivity through enhanced responsibility to the patients/users and a democratic discourse heeding the moral rights of the individual.^10^ In line with this, user involvement in research is argued through both utility and efficiency as well as through research ethics.^11^ Another rationale is that involvement in research is related to the recognition of the experiential knowledge as a contribution to theoretical and/or evidence‐based knowledge.^12^

A range of terms are used to describe the cooperation and the parties involved including consultation, participation, collaboration, involvement and partnership, to name a few.^13^, ^14^ These concepts have different meanings but are often used interchangeably.^14^ Terms used to refer to those involved in user involvement in health research include user, service user, patient, public, customer and consumer. These concepts trigger different associations depending on affiliations to different discourses; related either to ethical rights or to consumerism.^13^

Research has focused on different levels of user involvement^15^, ^16^, ^17^, ^18^ that have been defined and measured on a continuum ranging from a low to a high level of involvement.^17^ The linear hierarchical ladder of participation^18^ developed by Arnstein does not mention levels of participation as a process, but underlines it is possible to move up and down the rungs. Within this focus of research, information and consultation are described as the lowest level of involvement.^17^, ^18^ Collaboration is described as cooperation between researchers and users throughout the research process.^17^, ^18^ The highest degree of involvement is achieved through user‐led research where researchers are only involved when invited by users.^17^, ^18^

Knowledge development on user involvement in research is related to the question about which stages^19^ and extent^20^ of the research process users should be involved in. There are few research‐based descriptions of user involvement in health research as a process and it is not always evident in whether users have been involved in research projects.^21^

Reviews indicate that although there are both positive and negative experiences, descriptions indicate that users and researchers experience user involvement in research differently.^4^, ^21^, ^22^ Negative user experiences included concerns about not being heard,^21^ being marginalized within the research team and feeling uncomfortable.^4^ Positive user experiences included gaining a greater insight into research, a feeling of empowerment and satisfaction as a result of having contributed to something meaningful.^4^ Users also felt they were part of a team and socialized with others in similar situations. They gained a greater understanding of their illnesses and were brought up to date with the latest innovations in the field.^4^ Studies on the researchers, describe them to have multiple concerns. They feared sharing power and worried about the cost of the users' involvement, time required to develop working relations and the unrealistic goals of certain users.^4^ Positive aspects mentioned by researchers included an increased understanding of the issues and requirements from a community health perspective.^4^

Although literature indicates diverse challenges in the collaboration between users and researchers,^4^, ^20^, ^21^ existing health research literature focuses on the experiences of either users or researchers, advantages and disadvantages of involvement, how and when to involve users, and factors affecting the collaboration, little is known about the process of involvement itself. In other words, on how researchers and users interact when cooperating. To focus on the process of interaction implies not only paying attention to individual statements, but also to how other participants react to the statements. Bringing patient representatives and researchers together in an interview setting is not the same as observing the interaction in a “real” setting of collaboration in a research project. Still, bringing the two parties together will enable us to explore responses to statements and may provide an understanding of the process.

### 1.1 Aim

The overall aim of this study was to investigate experiences and collaboration between patient representatives and researchers in user involvement in health research.

We addressed the following two questions: (a) Which aspects of collaboration concerns patient representatives and researchers when they look back at research projects they have taken part in? (b) How do participants position themselves and each other through their descriptions of previous involvement projects?

### 1.2 Positioning theory

We consider user involvement as created and recreated through interaction processes between users and researchers. A closer look at the collaboration between researchers and users thus called for a theoretical framework that enabled us to focus on the interaction processes. We found positioning theory to be an appropriate tool for capturing this process.^23^ Positioning theory, as outlined by van Langenhove and Harré,^23^ is based on the idea that humans constantly position themselves within existing frames, in a process where positions, speechacts and storylines constitute a triad. Positions are created through the participants' understanding of reality in an ongoing and constantly changing process.^24^ During a conversation, the involved parties (researchers and users) assume various positions by expressing their own views as well as by responding (verbally or non‐verbally). We consider these expressions as speechacts that position the participants. Storyline is the story that emerges through positions participants ascribe and assume through speechacts.^23^ These three elements are mutually determinative. Positioning is a process of constructing social identities of oneself and others. Each position is constructed with various moral rights and duties, that determine what someone can or cannot say or do in any given situation and particular context.^23^, ^24^, ^25^

## 2 DESIGN AND METHODS

Grounded in a constructivist view, we consider focus group as a social space where participants co‐construct their views by sharing, acquiring and constructing knowledge.^26^ The choice of using focus group interviews was justified in our epistemological stand seeing focus groups as suitable space to study what participants are preoccupied with as well as the interaction between the two parties.^27^ The latter contributes to identifying the dynamics between the participants and the various positions they assume, reject and ascribe to themselves and to others.

### 2.1 Setting and data collection

To meet the study's aim, we needed data that captured the experiences of both users and researchers within a collaborative research, as well as the interaction between them. We were aware of an ongoing development project conducted by CHARM (the Research Centre for Habilitation and Rehabilitation Models & Services) that aimed to devise a model for user involvement in rehabilitation. To lay the grounds for participation in the development project, CHARM leaders had decided to invite researchers and patient representatives to discuss previous experiences from involvement in research through focus groups. Focus group interviews were seen as a way of gathering the views of the participants and to identify important points of involvement in the development of the model.

### 2.2 Focus group interviews

CHARM conducted two focus groups in November 2016 at the University of Oslo, Norway. The user representative in charge of the CHARM project included a majority of patient representatives in the focus groups in order to reduce power imbalances between researchers and patient representatives. Invitations to participate in the focus group interviews were sent to patient organizations that were members of the Norwegian Federation of Organizations of Disabled People or the Norwegian Forum of Disabled Peoples Organizations. The patient representatives represented different organizations and had not previously collaborated with the researchers. The participants had varying experiences with involvement in research. (While some users have been involved in steering boards, panels and as co‐researchers others were new to the project). Researchers were recruited through the snowball method^28^ by the researchers in the CHARM group.

Both focus group interviews were moderated by the same researcher from the CHARM group. The first group consisted of seven patient representatives and three researchers, and the second group consisted of seven patient representatives and two researchers. Both interviews lasted 2 hours and 15 minutes with a 15‐minute break after every 45 minutes. CHARM used a semi‐structured interview guide, which focused on experiences of involvement in research, to guide the interviews (Appendix S1).

At the beginning of each session, the moderator presented the topics of that particular session. During the sessions, the moderator regularly summarized the information presented by the participants and formulated questions based on the information provided. The interviews were tape recorded.

We approached the leaders of CHARM and requested to use their data for our research purposes. We saw the data as an opportunity to take advantage of the fact that both parties were present and to explore how participants positioned themselves and others in their descriptions of their experiences.

Information of our study and requests to use the data were sent to each participant to obtain informed consent.

### 2.3 Data analysis

The audiotapes of the interviews were handed over to us and it was transcribed verbatim by the first author. From here on, we refer to the texts as “our” data. As we (the authors) had not participated in the focus group, we listened to the tapes several times and took notes on the verbal interactive process.^29^ All authors read the transcripts, and for the next step of analysis, the files were transferred to Hyper Research (Researchware Inc, 2014). The next step of the analyses focused on which factors the participants raised as important and these factors were transferred to codes in Hyper Research. The codebook allowed us to identify which codes had similar meanings and to reorganize the codes accordingly. Thereafter, we disconnected Hyper Research. The codes were further processed by moving back and forth between speechacts, research questions and previous knowledge to generate categories. All authors participated in analysis meetings. We focused on how participants' talked about user involvement^27^, ^30^, ^31^ by identifying speechacts. In the next step, we identified positions, both ascribed and assumed through these speechacts (Appendix S2). The different positions expressed by speechacts lead us to two storylines. To identify these positions, we also focused on what we considered differences in the responses between representatives and researchers.

The researcher from CHARM who conducted the interviews reviewed the analysis and provided valuable feedback. The involvement of users in this study is described in Appendix S3.

We will further use the term user when referring to patient representatives from the focus groups.

### 2.4 Findings

The focus group discussion revolved around different storylines that were portrayed in an intertwined and interdependent way. The different positioning of the two parties was related (directly/indirectly or consciously/unconsciously) to different responsibilities, rights and duties in the research process. However, users and researchers were preoccupied with different aspects of the topics.

Within the focus group, all participants replied, but researchers primarily assumed passive positions and reacted in a mostly nonresponsive way to the users' statements, whereas users assumed active positions by expressing their wishes and needs as reflected in numbers of quotes. In the following, we will present two storylines: Status and knowledge and Being a partner or not. Each storyline includes the participants' descriptions of previous experiences, followed by a description of the interaction in the focus group.

### 2.5 Status and knowledge: two different worlds

This heading stems from a statement by one user: *We come from two different worlds *(User 2). The statement might be an indication of how disparate users perceived the different forms of knowledge. This difference is described as related to “levels”:

I can see that we have a considerable responsibility ourselves (to be active in the process), but I am not sure how to manage that responsibility, possibly because there is a mismatch between the levels. (User 4)

This statement might suggest that users feel powerless and find it difficult to assume responsibility in the research process and consequently assign this responsibility to the researchers.

Although users assumed their experience was an important asset, they also acknowledged that the researchers' knowledge had a higher status (and thus more power) than their own:

Sometimes, I think it can be quite demanding to be a patient representative in a research project because there is a mismatch between competences, and it rarely happens that competence based on experience is highly valued; so when you meet researchers with substantial theoretical knowledge, it is rarely on even terms. (User 1)

Even though the users positioned themselves as important, they also positioned the researchers as decision‐makers with the power, duty and responsibility to lead the process:

The user's experience is really important, I completely agree, but in the end, the researcher is the expert and the one who does the job, and we need to trust that they know what they are doing. (User 6)

Some statements indicated that feeling powerless and having lower status was difficult:

When we talk of social status and my experience is that you feel like a trivial part of the research panel, together with all the great gurus who are accustomed to expressing themselves in great detail and who generally take up a lot of space. (User 3)

Users expressed a wish to receive more scientific knowledge and education about the research process, which simultaneously leads to a higher status, and this seemed to be an important factor:

I agree with those of you who mention the importance of increasing our status, and I am sure that will happen eventually. (User 14)

During focus group discussions, the researchers assumed passive and silent positions and did not mention their own theoretical competence. Users underlined their experience as an important contribution to the research, and they mentioned their experiences several times, both implicitly and explicitly.

In discussions of power imbalance, the researchers' use of language was highlighted as an important factor.

Researchers and the PhD candidates need to try to speak in layman's terms and not talk for each other but for us, so we can participate. (User 2)

Researchers did not respond directly to the users' statements, but they expressed interest in the users' experience‐based knowledge.

I wanted input from those who represent the patient group. Through various channels this patient representative has contributed to boost recruitment, in addition she has helped define what should be researched through planning of the project. (Researcher 3)

Although statements such as the one above showed the researcher's respect towards the users, there were also statements where the researcher assumed a position that carries the right/responsibility, and therefore, also the power to decide the user's degree of involvement:

What if the user is useless? Can we then ensure the project has an alternative solution to secure steady progress despite this? What we are doing, seen from the researcher's perspective, is to ensure that we are not left with someone who is a nuisance in meetings or who is useless and scares off any researcher for the time remaining. (Researcher 5)

Useless and nuisance are strong terms. However, the user responds to this statement not by refuting the idea of a useless user but by pointing out that this is something that could occur in any situation:

With regard to a useless user, in one way, that is the downside of volunteerism. Regardless of where we are, whether it is an election to a board or a user to a board, we run the risk of encountering people who do not do their jobs…. Of course, at the same time, one needs to be a little strategic and good at identifying the right people. (User 4)

The user assumes a defensive position while simultaneously noticing that one should have a plan (be strategic) and thereby agreeing that there is such a thing as a “useless” users.

### 2.6 Being a partner or not

Being positioned as partners was considered important, buts users considered this position difficult to attain. *In the end, it is the researcher who makes the decisions* (User 5). Users do not position themselves as having the right to make decisions in the project, this interpretation is supported by the following:

Yes, user involvement can be many things, and it could be that it is not possible to meet one another on even terms. (User 9)

As demonstrated power imbalance may be an important obstacle in involvement.

Indications of being a partner were described as important. A user mentioned he had not been invited to a study trip and felt he had been treated wrongly. This viewpoint was supported by other users. Participating in the same events as the researchers seemed to strengthen the feeling of being part of the team. Another aspect was the feeling of being alone:

It is like being a hostage, right, when you join the various projects and a bunch of colleagues are sitting around the table and a stranger like you walks in. I think they have a responsibility to take care of that stranger. As you mentioned, it is important to be taken care of with regards to what you promise to contribute and the things you dare to say. (User 5)

The statement *like being a hostage* can be interpreted as the feeling of only having been included because of a regulation that requires it as “icing on the cake.” However, others refuted this position by demonstrating their important contribution:

My research partner said that I am different; I asked entirely different questions than she did and collaborated on the design of an interview guide for people with problems, which is essential. (User 3)

The notion of getting something back (positioning the researchers as individuals with a duty to give something in return) suggested social involvement, acknowledgement and having knowledge that could benefit their group of patients, thus legitimizing a claim of partnership.

We also demand that when they have reached a certain milestone, it should be boiled down to small articles in Norwegian for the layman—articles we would like to include in our magazine. (User 2)

Researchers did not comment on the users' wishes directly but did recognize the importance of good cooperation.

I explained that I would create a summary that I will send to all of you and that you can publish on your website, but it is very difficult to prioritize. (Researcher 2)

Researchers mentioned a lack of time and money as the most common reasons for not including the users in every stage. The users also considered time and money a challenge but noted that careful planning was required from the beginning.

Within the focus groups, the users were also more active than the researchers. While the users were preoccupied with being seen and heard, the researchers were preoccupied with questions of time and money.

Our findings indicate that the users positioned themselves as a group, as opposed to the researchers, who spoke as individuals.

In the next excerpt, one user adopted the position of being resourceful and able to give the researchers advice on how to recruit users with few resources:

Who we see are those with many resources, the resourceful ones who can take on the task of being a representative. You will not find those who really have problems and who do not master their day‐to‐day lives. They do not have the energy to come and meet you; if that is the group you want, you need to go to people's homes. (User 3)

Another topic discussed in the group was the users' desire to participate in social events. The researchers agreed to this as long as the number of events was restricted:

It is really important that you include (users for) a dinner, not every time. I think that is a part of being a project leader, and I also need to spend time on it, and of course, I know that I will eventually follow through because the quality will be better. (Researcher 1)

The researcher assumes a position of self‐reflection in supporting the statements from the users in this matter. This statement also indicates that she perceives it to be the duty of a project leader. The researcher provides something with the hope of getting something in return: better quality.

In the next excerpt, one researcher presents her reflections of why involvement can be difficult.

There are several reasons why the users are often considered as “icing on the cake”; sometimes, it could be because the researcher believes it is a hassle, and we simply need to have them to fulfil a criterion. I think often it is simply uncertainty; one does not quite know how this should be done since one does not have that much experience. (Researcher 1)

The fact that the researcher employs the term researcher rather than I suggests she does not relate to the situation, but then she suddenly uses the pronoun we, suggests that she is talking about something she has experienced personally.

The next statement sums up a general impression that the users and researchers are not clear on their roles and what is expected of them.

The most important thing to do in the beginning should be to clarify what is expected of the users. (User 8)

## 3 DISCUSSION

By bringing together users and researchers in a focus group (as opposed to separate interviews) to discuss user involvement in research, we created a setting that to a certain degree, resembles reality. Employing positioning theory enabled us to identify the positions adapted in the encounter. The findings illustrate how ideals of user involvement are played out at the microlevel and how these ideals reflect and relate to different perceptions of user involvement. Our findings show that researchers are positioned on a higher level of involvement^18^ and thus have more decision‐making power than users.

Although interconnected and overlapping, three themes stood out as important in the discussion and are related to power through constant implicit and explicit negotiation of positions.

1. Users were preoccupied with the assumption that different knowledge yields different status, as pointed out earlier.^32^ The desire to capture the experiences from “the ones who wear the shoes” may carry and lean on a democratic discourse heeding the users' informal knowledge and a discourse of effectivity searching to reduce costs and increase users' responsibility. There might be tensions between these discourses, but also between expert knowledge and evidence‐based practice carried by researchers.
2. Conflicting positions also affect the views of equity and make statements of equity appear to be an important but blurred factor, leaning on a strong correlation between knowledge and thus power and status. The users in our study claimed equal recognition of both types of knowledge, but they did not feel that their experiential knowledge was positioned as equally valuable to science‐based knowledge which is in line with previous research.^33^, ^34^ Despite the fact that users claimed an equal recognition, they also asked to receive more science‐based knowledge to smooth out the inequality between positions. Scientific knowledge is considered by the users to lead to a higher status and a stronger position, which in turn makes it easier to act as equal partner.
3. In the discussion of knowledge and thereby also status and power, there was an ongoing negotiation about partnership. According to our results, being positioned as a partner seems to be important to users, but different rights and duties made a partnership difficult to achieve.

Power issues are known within user involvement in research,^4^ and our study shows that different rights and duties influence how researchers consciously and unconsciously assume and are ascribed positions with more power. Using Arnsteins ladder^18^ one could state that users and researchers are positioned on different rungs throughout the process but with a tendency that researchers assume but also are ascribed positions on a higher level of participation.

On one hand, users want to be independent and equal partners, on the other hand, they are dependent on the researchers. The conflict between responsibility versus dependency may be seen as yet another reflection of the dichotomous ideals of dependency (“being the helped” vs independently “being the customer”) carried within the politics of involvement.

In the following section, we will further describe how the two parties interacted. Although bringing together the two parties was a way of imitating a real setting, research projects generally include more researchers than users, and in this respect, our study deviated from reality. The fact that there were a majority of users in our focus group might explain why they were more active in voicing their opinions, indicating that numbers count. It is not uncommon for the party with less power to emphasize its rights and the party with more power to emphasize its duties.^25^

Both users and researchers seemed to feel insecure about their roles. Shared insecurity might lead to a shortcoming in discussions of different perceptions and desires. Conflicting views about rights and duties can arise when the rules have not been clearly defined.^23^ Understanding each other's needs and goals is described as an important factor in a collaboration,^35^ and we agree with Goldman and Schmalz^36^ that clear, transparent expectations are fundamental to a successful collaboration. Users in our groups experienced insufficient communication within previous projects.

In the focus group, the two parties responded to each other's concerns to a small extent, This might suggest that they were not preoccupied with the same issues or that they felt uncomfortable commenting on the other party's views. This pattern might reflect “real” research collaboration.

While the position of insecurity was similar in both groups, the way in which they interacted suggested differing positions: active and passive. The users were quite active during the discussions. However, their own descriptions of previous research projects suggested they had assumed a passive and powerless role. The two different behaviours can have several explanations and might be the result of the situation—or perhaps a result of the fact that there were a majority of users in the focus group. Tan and Moghaddam^37^ state that dominant groups have more legitimate voices and that they are more “entitled” to speak and to be heard.

The relative silence of the researchers could be interpreted as a gesture of respect or suggest that they felt uncomfortable commenting on the users' statements. The relatively active role of the users' during the focus group interviews could also suggest that they strove to obtain acknowledgement of their felt subordinate positions. However, this position was combined with an opposite claim, where users positioned themselves as having the right to be heard, implying the researchers' duty to listen. The researchers' silence may be interpreted as supportive, as it avoided not disturbing the users when they expressed their opinions, desires and frustrations. Previous studies have indicated that although the researchers' silence allow the users to be heard, the silent acceptance could also cause users to feel infantilized and frustrated about not receiving answers to their questions.^38^

One may state that democratic discourse would position users to have the right to influence the development of knowledge and thus be active, but simultaneously, they have a duty to contribute to the efficiency of the research process in accordance with the managerial discourse and not be a bother by taking up time. Involvement thus becomes a result of different discourses carrying diverse values, expectations and requirements.

### 3.1 Strengths and limitations

An important limitation of this study is that none of the authors participated in the focus group interviews, limiting findings to the speechacts and omitting non‐verbal interaction. We searched to compensate by gathering useful information from the moderator of the interviews and presenting our findings to the participants.

By analysing interaction in the focus groups, we were able to understand how positioning processes evolved during discussions and created positions that might shed light on some of the challenges of interaction described in the literature.^4^, ^22^, ^39^ Another limitation is the limited number of participating researchers; however, the limited number could also be seen as a strength. Having a majority of users might strengthen their sense of security, which in turn allows them to speak more freely.

## 4 CONCLUSION

The positions that users and researchers assume and ascribe throughout the process are constantly changing. Different positions in the form of dissimilar rights and duties create power differences and these stand out as barriers in the collaboration process. The different positions adopted can be related to conflicting ideals within user involvement. The fluidity and constant change in positions may veil that the researchers tend to assume and be ascribed positions with more power both with regards to knowledge, status and partnership. These different positions might challenge an equal collaboration between users and researchers and it seems that the ideal of coproducing research is hard to reach. The relationship between equity in user involvement in research and power needs to be studied further to understand how dilemmas, contradictions and paradoxes in the research process evolve.

## ETHICAL APPROVAL

We obtained ethical approval from the Norwegian Centre for Research Data.

## CONFLICT OF INTEREST

The authors declare that there is no conflict of interest.

## AUTHOR'S CONTRIBUTION

All authors contributed in conducting this study and writing the article. SS transcribed the interviews. SS, ICF and IRK read the interviews and conducted the analysis. All authors read and approved the final manuscript.
