# Supplementary material for: Coming from two different worlds—A qualitative, exploratory study of the collaboration between patient representatives and researchers
Source: Health Expect. 2019 Feb 18;22(3):496–503. doi: 10.1111/hex.12875 (PMC6543145; doi:10.1111/hex.12875)
Supplement: Supplementary file 1 [file HEX-22-496-s001.pdf]

Appendix 1: Interview guide

1) Describe previous research projects you have participated in.

2) How did you experience their participation?

(Were there some special aspects of participation that were positive/ negative or difficult?)

3) Please describe how you participated in the project. (Which roles did you have?)

4) How were the roles defined? (To what extent did you discuss your participation and the collaboration process with the researchers? Were you given the opportunity to define your own participation?

5) What was the most challenging aspect of your participation in the research project?

6) What was the most interesting or rewarding aspect of your participation?

7) What is important to take into consideration when planning user involvement in research?

Time, location?

Financial compensation?

Role clarification?

Training?

Anything else?

8) What are your thoughts regarding financial compensation for user involvement?

9) Do you think financial compensation has an effect on who participates in such projects and which roles they are given?

10) Are there any important aspects of user involvement which have not been covered?
