# Supplementary material for: Coming from two different worlds—A qualitative, exploratory study of the collaboration between patient representatives and researchers
Source: Health Expect. 2019 Feb 18;22(3):496–503. doi: 10.1111/hex.12875 (PMC6543145; doi:10.1111/hex.12875)
Supplement: Supplementary file 2 [file HEX-22-496-s002.pdf]

Appendix 2: Analysis inspired by positioning theory

Participant	Speech-act	Position of the participant	Rights	Duties	Ascribing positions to the other(s)	Storyline
**User**	Feeling like icing on the cake	Icing on the cake	None	To participate when it suits the researcher	The researcher has the power	Being a partner or not
**Researcher**	It is important to go out for dinner	The carer	To manage the social aspect of the involvement process	To get to know the users and take care	The users need to be taken care of	Being a partner or not
**Researcher**	I explained that I would create a summary that I will send to all of you(the users) and that you can publish on your website but it is very difficult to prioritize	A position with duties and responsibilities	To decide when the users should be included	Stick to deadlines	The user has to have an understanding for my challenges	Partner or not
**User**	it is important to be taken care of with regards to what you promise to contribute and the things you dare to say	The helpless	To be taken care of	To contribute	The researcher has to take care of my	Partner o r not
**User**	you feel like a trivial part of the research panel, together with all the great	Positioned as not confident	None	To be present at	The researcher takes the	Status and knowledge

Appendix 2: Analysis inspired by positioning theory

	gurus who are accustomed to expressing themselves in great detail and who generally take up a lot of space			the meeting	leading positions	
**User**	Researchers need to adapt their language so that we can understand and participate	Dependent position	The right to participate<br>To claim form the researcher to adapting their language	None	Researchers have to adapt themselves to the users	Status and knowledge
