# Supplementary material for: Coming from two different worlds—A qualitative, exploratory study of the collaboration between patient representatives and researchers
Source: Health Expect. 2019 Feb 18;22(3):496–503. doi: 10.1111/hex.12875 (PMC6543145; doi:10.1111/hex.12875)
Supplement: Supplementary file 3 [file HEX-22-496-s003.pdf]

Additional file 3

User involvement in this study

Recruitment	A user representative from CHARM was involved in designing the invitation letter.
Data collection	The same user representative contributed in planning the focus group interviews. The user representative preferred being an observer instead of conducting the interviews.
Writing of manuscript	Another user was involved to critically read the manuscript and discuss its contents, since the user representative from CHARM did not have time to do so.
Dissemination	We informed participants from the focus groups of the preliminary results. Participants will receive the article when approved.
